# Finesse and Fire, Creativity and Combustion

**DOI:** 10.3201/eid2504.AC2504

**Published:** 2019-04

**Authors:** Byron Breedlove

**Affiliations:** Centers for Disease Control and Prevention, Atlanta, Georgia, USA

**Keywords:** art science connection, emerging infectious diseases, art and medicine, about the cover, Finesse and Fire, Creativity and Combustion, Untung Yuli Prastiawan (aka Wawan Geni), The Little Time, mosquito coils, vector-borne infections, malaria, insect repellents, Indonesia

**Figure:**
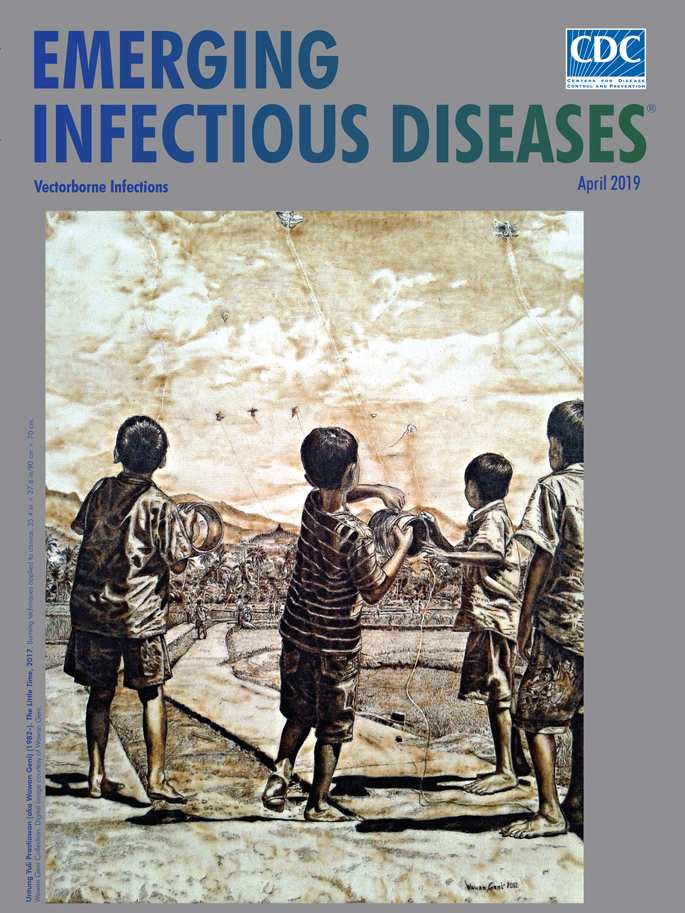
**Untung Yuli Prastiawan (aka Wawan Geni) (1982–). *The Little Time*, 2017.** Burning techniques applied to canvas, 35.4 in × 27.6 in/90 cm × 70 cm. Wawan Geni Collection. Digital image courtesy of Wawan Geni.

The Republic of Indonesia is the world’s fourth most populous country, the largest archipelago nation, one of the world’s most biodiverse regions, and the country with the most active volcanoes. Vectorborne infections spread by mosquitoes pose a significant health burden for this equatorial nation. For Indonesia, World Health Organization data indicate that the incidence of malaria in 2015 was 26.10 new cases of malaria per 1,000 population at risk, 41st among all countries. Indonesia also has one of the highest burdens of dengue fever, and Japanese encephalitis and chikungunya are endemic there. Small wonder that a May 2014 article in the *Business Nikkei Asian Review* reports, “Some 4 billion mosquito coils are sold annually in the tropical archipelago.”

Indonesian artist Untung Yuli Prastiawan, also known as Wawan Geni (“geni” in Javanese means fire), has purchased more than his fair share of those coils―but not for warding off mosquitoes. He uses them to create detailed works of art. Prastiawan is from Magelang in Central Java, Indonesia, and is a graduate of the Indonesian Institute of the Arts. He started his career as an oil painter. In 2003, he had the idea of creating images by burning them on drawing paper. Initially, he worked with incense, burnt sticks, and blackened firewood as his “brushes.” Dissatisfied with those results, he continued experimenting and discovered that the glowing embers from mosquito coils and cigarettes accorded him the precision and control he was seeking from a combustible medium. His unusual painting technique has garnered Prastiawan many awards, including special recognition by the Indonesian World Records Museum in 2006.

Videos available on the Internet offer a glimpse of Prastiawan at work. He starts with a blank canvas or sheet of thick drawing paper, a stack of mosquito coils, and numerous cigarettes piled pell-mell. After soaking the coils in water, Prastiawan snaps them apart, lights a section, and darkens the canvas by using the smoldering tip of the coil like a charcoal pencil and the cigarette like a miniature blowtorch on the tip of the coil. As he blows on an ember, he balances finesse and fire until detailed sepia-tinged shapes and images emerge. The artist cautiously and continually gauges how much heat he is applying and even heeds subtle shifts in wind direction throughout the process.

Completing each drawing, depending on its size and complexity, typically requires months of work, thousands of exhales, and, according to the artist, typically around 18 packages of mosquito coils. In a sense, Prastiawan’s unusual approach to art embodies the notion of surrealist painter and sculptor Joan Miro that “the works must be conceived with fire in the soul but executed with clinical coolness.”

According to Prastiawan, “The Little Time,” this month’s cover image, is “about my daily life when I was a child” (pers. comm., 2019 Feb. 14). In this drawing, he pays homage to his traditional culture by portraying a group of barefoot boys unfurling their kites and gazing toward the sky. More children can be seen near the fence that separates the field from a strip of forest. The temple visible on the hilltop before a low range of mountains is actually Borobudur, the 9th century Buddhist temple in Magelang that was built from volcanic stones and designated a UNESCO World Heritage Site. Prastiawan fills the canvas with delicately rendered textures of trees, grasses, and clouds, the interplay between light and shadow, and even a miniature scene on the pant leg of the boy on the right. “The Little Time” is an idyllic image, free from irony, save the fact that the artist said that it took him 5 months to finish.

It seems a safe assumption that Japanese entrepreneur Eiichiro Ueyama, who invented the spiral mosquito coil, never envisioned it could also be an instrument for creating art. For more than a decade, Ueyama persistently experimented with mixtures of pyrethrum powder and other ingredients and with various manufacturing techniques but failed to create a long-lasting combustible insect repellent. Early prototypes included sticks and bars, neither of which worked as he envisioned. His wife, Yuki, suggested the spiral shape, and Ueyama then spent 7 years perfecting the product, finally marketing the first coils in 1902.

Despite their widespread use, mosquito coils have not been demonstrated to prevent malaria infection, though evidence indicates that they reduce nuisance bites from mosquitoes. To decrease the global health burden of disease caused by vectorborne infections, researchers and public health experts continue to develop and evaluate inventive approaches, including enhanced surveillance, smart mosquito traps, biotechnology tactics, and new and improved vaccines. Those endeavors will be aided by the blend of creativity, finesse, and tenacity exhibited by Prastiawan in his novel, painstaking approach to his art and by Ueyama in his unrelenting drive to create a viable mosquito repellent.
